# Henry Earle

**Published:** 1838-04

**Authors:** 


### OBITUARY.

### HENRY EARLE, F.R.S.

*Surgeon Extraordinary to the Queen.*

DIED, on the 18th January, 1838, at his house in George street, from an attack of fever, HENRY EARLE, Esq., Surgeon of St. Bartholomew's Hospital, and one of the most eminent and most respected members of the profession. Mr. Earle was the third son of Sir James Earle, by a daughter of the celebrated Percival Pott, and was born June 28th, 1789. He manifested, very early in life, a strong predilection for the profession of surgery; and was therefore apprenticed to his father, at the age of sixteen. At the early age of nineteen, he held the appointment of house-surgeon at St. Bartholomew's Hospital; and in 1811, being then twenty-two years of age, he commenced practice. He very early distinguished himself among the pupils of the hospital, by the invention of a bed for patients suffering from fractures of the lower limbs and diseases of the spine; for which he received, in 1812, a reward from the Society of Arts, and which he afterwards so much improved as to induce the Society, in the year 1821, to confer on him a second and larger prize. In 1813, the College of Surgeons awarded to Mr. Earle the Jacksonian prize, for an Essay on the Diseases and Injuries of Nerves; an honour which was shortly afterwards followed by his election as surgeon of the Foundling Hospital, on the death of Mr. Ramsden. In the year 1815, a wider field was opened for the display of his talents and the exercise of his activity and zeal, by his election to the assistant surgeoncy of St. Bartholomew's Hospital; in which situation he remained until 1827, when he was promoted to the office of surgeon, which had become vacant by the resignation of Mr. Abernethy. Ever alive to the interests of the students, Mr. Earle immediately availed himself of his new position to supply a deficiency which had been long felt by them, in regard to clinical instruction: he instituted a gratuitous course of clinical lectures, (the first that had ever been given at that hospital;) and these he continued to deliver, on all suitable occasions, during the remainder of his life: a boon which was very highly esteemed by the pupils of the hospital.

Mr. Earle was for several years a Member of the Council of the Royal College of Surgeons; and, in 1833, gave his first course of lectures, as Professor of Anatomy and Surgery, in the College. He held the office of President of the Royal Medical and Chirurgical Society, from March, 1835, to March, 1837, having previously acted as Secretary and Treasurer for many years. On the accession of the present Queen, he was nominated one of the Extraordinary Surgeons to her Majesty.

In all the relations of private life, Mr. Earle was most exemplary: he was an affectionate husband, an indulgent father, and a sincere and constant friend. His public duties at the Hospital were performed with such ardour and exactness, that to a casual observer, the labour might have seemed a source of direct enjoyment to him, instead of being the result of a sense of duty. To all his private patients Mr. Earle was no less the friend than the medical adviser, and beloved even beyond the usual measure of regard accorded to those characters.

Mr. Earle has left eight children, (two daughters and six sons) who, we are happy to hear, are very comfortably provided for.

The following is a list of the principal writings of Mr. Earle:

*In the Medico-Chirurgical Transactions,*

Vol. iii. Feb. 1812.—Case of diseased Testicle, accompanied with disease of the Lungs and Brain.

Vol. v. May 10, 1814.—On Contractions, after burns or extensive ulceration.
Vol. vi. Jan. 3, 1815.—On the Use of Nicotiana in retension of Urine.
Vol. vii. Feb. 20, 1816.—Cases and Observations, illustrating the influence of the nervous system in regulating animal heat.
Vol. x. Dec. 21, 1819.—On Affections of the Meatus auditorius externus.
Vol. xi. March 28, 1820.—On the Danger of Extracting large Calculi, with a description of an instrument for breaking down stones of considerable magnitude.
June 20, 1820.—On Renal Calculi.
Vol. xii. April 2, 1822.—Cases of ununited fracture of the Humerus, treated by seton and caustic.
Dec. 10, 1822.—On the Influence of Local Irritation in the production of diseases resembling Cancer.
Jan. 14, 1823.—On Chimney Sweepers' Cancer.
Vol. xiii. June 19, 1827.—On Paraplegia.
Vol. xiv. April 14, 1835.—Observations on Fractures of the Bones of the Pelvis.

*In the Philosophical Transactions,*

April 12, 1821.—On the Re-establishment of a Canal in the place of a portion of the Urethra, which had been destroyed.
April 25, 1822.—On the Mechanism of the Spine.

*Separate Publications:*

Practical Observations in Surgery. 8vo. London. 1823.
Two Lectures on the Primary and Secondary Treatment of Burns. 8vo. London. 1832.

